# Generative AI as interactional infrastructure for meaning-centered care in later life

**DOI:** 10.3389/fpsyt.2026.1883962

**Published:** 2026-06-16

**Authors:** Bing Chen, An-Yue Jin

**Affiliations:** 1 Third Affiliated Hospital of Wenzhou Medical University, Wenzhou, China; 2 Wenzhou Central Hospital, Wenzhou, China

**Keywords:** digital mental health, dignity therapy, generative artificial intelligence, large language models, life review, meaning-centered care, older adults, public mental health

## Abstract

Generative artificial intelligence (GenAI) and large language models are rapidly entering mental health research and service delivery, yet their dominant use remains symptom-centric, emphasizing screening, classification, triage, and risk detection. For older adults, mental health is often inseparable from existential concerns: loss of social role, disrupted continuity of self, loneliness, diminished dignity, and questions of legacy. This perspective argues that GenAI should not be conceptualized as an autonomous substitute for clinicians, nurses, social workers, or family caregivers. Instead, it may be better understood as an interactional infrastructure for meaning-centered care in later life. Drawing on meaning-centered psychotherapy, dignity therapy, life review, gerotranscendence theory, care ethics, and implementation science, we propose a Sensing-Narrating-Connecting-Governing framework. In this model, multimodal AI systems help detect existential and relational cues, support life-review conversations, co-construct dignity-preserving narratives, connect older adults with human care networks, and operate under explicit safeguards for privacy, hallucination, dependency, crisis escalation, and cultural adaptation. The proposed framework shifts evaluation from model performance alone toward existential well-being, dignity, continuity of self, therapeutic alliance, equity, and workflow integration. We conclude that GenAI may contribute to public mental health only when deployed as a bounded, human-supervised, culturally responsive layer of relational augmentation rather than as a replacement for human presence.

## 1 Introduction

Generative artificial intelligence (GenAI), including large language models (LLMs), is changing the technical possibilities of digital mental health. Unlike earlier discriminative systems that primarily classified symptoms or predicted risk, GenAI can produce conversational responses, summarize complex narratives, generate psychoeducational content, and integrate text, speech, image, and behavioral signals into interactive applications. In psychiatry and public mental health, this has stimulated interest in chatbots, digital triage, clinical decision support, simulated patients, risk monitoring, and AI-augmented psychological interventions. These developments are especially relevant for aging societies, where mental health systems face rising demand, workforce shortages, fragmented community services, and increasing numbers of older adults living with chronic illness, cognitive decline, bereavement, loneliness, or functional limitations.

Yet the prevailing direction of AI-enabled mental health remains narrow. Much of the field is organized around symptom detection, diagnostic classification, crisis prediction, and service efficiency. These aims are important, but they do not exhaust the mental health needs of later life. For many older adults, psychological distress is not simply a set of symptoms; it is entangled with questions of meaning, dignity, identity, dependency, regret, mortality, and legacy. The late-life question is often not only “How severe is the depression?” but also “What still gives this life coherence?”, “How can past losses be integrated?”, “Who will remember what mattered?”, and “How can dignity be preserved when autonomy declines?”.

This perspective argues that GenAI should be repositioned in relation to these existential concerns. Rather, GenAI may function as an interactional infrastructure for meaning-centered care: a bounded, human-supervised layer that helps elicit life stories, organize memory, identify meaning-related distress, support dignity-preserving narrative work, and connect older adults to clinicians, nurses, social workers, family members, and community resources. Such a shift moves the field from human replacement to relational augmentation.

The argument is deliberately conceptual rather than technological determinist. We do not assume that current LLMs are ready to conduct psychotherapy, nor that multimodal sensing can infer meaning without human interpretation. Instead, we suggest that the next stage of AI in older adult mental health should be judged by a different question: whether these systems can help care organizations become more attentive to the biographical, relational, and existential dimensions that are often lost in overstretched services. This question is well aligned with the public mental health orientation of scalable, preventive, and equitable care, but it also demands stronger boundaries than ordinary consumer-facing chatbot design.

The term interactional infrastructure is used here as a conceptual synthesis rather than as a claim of priority over adjacent frameworks. Digital health frameworks from implementation science have already emphasized intervention development, user engagement, behavior change, feasibility, and real-world evaluation (1, 2). Human-robot interaction and assistive-technology research with older adults has also shown that technologies can support companionship, independence, safety, and aging in place while raising concerns about trust, dependency, and relational quality (3). The contribution of the present framing is to apply these implementation and relational insights to a specific GenAI problem: how systems that generate, summarize, and circulate personal narratives can support meaning-centered care without replacing the human listener or obscuring accountability.

## 2 The existential blind spot in AI-enabled mental health

Meaning-centered approaches have a long intellectual and clinical history. Frankl’s logotherapy emphasized the will to meaning as a fundamental human motivation (4). Butler’s life review theory framed reminiscence in later life not as mere nostalgia but as a developmental process through which older adults integrate their lives, resolve conflicts, and preserve continuity of self (5). Tornstam’s theory of gerotranscendence further suggested that aging may involve a shift from material and role-based identities toward more cosmic, relational, and transcendent forms of meaning (6). In clinical contexts, meaning-centered psychotherapy and dignity therapy have translated these ideas into structured interventions for patients with advanced illness, caregivers, and palliative care populations (7–10).

These traditions are highly relevant to old-age mental health. Loss of role, bereavement, chronic disease, institutionalization, and social isolation can threaten a person’s sense of value and continuity. Conversely, life review, dignity-conserving care, intergenerational communication, spiritual reflection, and narrative integration may support resilience even when cure, functional recovery, or full independence is not possible. Meaning-centered care therefore shifts attention from symptom reduction alone to the preservation of personhood.

The empirical literature on reminiscence and life-review interventions offers a more relevant starting point than the general chatbot literature alone. Reviews and meta-analytic work suggest that structured reminiscence and life-review interventions can improve depressive symptoms, life satisfaction, and psychological well-being for some older adults, although effects vary by intervention format, population, facilitator training, outcome measure, and follow-up duration (11, 12). This evidence supports the clinical plausibility of narrative and meaning-oriented work, but it does not by itself validate AI-delivered intervention. It indicates that the active ingredients of life review, including autobiographical recall, emotional processing, social sharing, and integration of difficult memories, must be preserved rather than reduced to automated prompting.

Current AI mental health systems rarely reflect this orientation. Recent work on LLMs in mental health highlights rapid growth in applications, but many systems remain concentrated on diagnosis, risk detection, symptom assessment, or general counseling support (13). Broader critiques have questioned whether symptom-centric AI paradigms can adequately capture the complexity of mental disorders (14, 15). Multimodal systems using speech, language, facial expression, movement, and digital behavior are also being explored for depression, cognitive impairment, or other clinical signals (16, 17). These efforts are valuable, but they risk creating an existential blind spot: AI systems become increasingly capable of detecting distress while remaining poorly equipped to understand what makes a life meaningful to the person experiencing that distress.

This blind spot is particularly consequential for older adults. An AI system that detects sadness in an older person’s voice may identify risk, but it may miss the biographical meaning of that sadness: an anniversary of loss, unresolved guilt, fear of becoming a burden, or concern that one’s life story will disappear. Similarly, a chatbot may produce supportive phrases without helping the person connect memories, values, relationships, and future-facing purposes. In meaning-centered care, the relevant unit of intervention is not only the symptom but the story.

The distinction also changes what should count as innovation. A technically advanced system that predicts depressive symptoms more accurately may still leave untouched the existential concerns that sustain suffering. By contrast, a modest tool that helps an older adult articulate what should be remembered, what remains unfinished, or what forms of connection still matter may have genuine clinical and relational value. For this reason, meaning-centered AI should not be evaluated only as a diagnostic instrument. It should also be evaluated as a mediator of recognition: whether it helps previously unheard experiences become visible to the older person, the family, and the care team.

## 3 Why meaning-centered care cannot be reduced to chatbot support

It is tempting to imagine that an empathic chatbot could deliver meaning-centered care simply by asking reflective questions. This would be a mistake. Meaning-centered care is not a content module that can be appended to a digital interface. It is a relational and ethical practice grounded in recognition, witnessing, and the co-construction of meaning.

Dignity therapy illustrates this distinction. Its therapeutic value is not only that patients answer questions about important memories, roles, and messages for loved ones. It also depends on the presence of a trained listener, the editing of a generativity document, and the recognition that the person’s words deserve preservation (8, 9). Life review similarly depends on the emotional pacing and interpretive context in which memories are revisited. Meaning-centered psychotherapy is not merely a sequence of prompts about values; it is a therapeutic process that helps people locate sources of meaning despite suffering, limitation, and uncertainty (7, 10).

GenAI can support parts of this process but cannot own the whole process. It can help generate prompts, summarize narratives, detect recurring life themes, produce draft legacy documents, translate personal stories into accessible formats, and facilitate communication between older adults and care teams. However, it cannot carry moral responsibility for clinical judgment, crisis response, informed consent, or the relational obligations that attach to human care. The appropriate question is therefore not “Can AI provide meaning-centered care?” but “Which components of meaning-centered care can GenAI augment, under what safeguards, and with what forms of human accountability?”.

This distinction matters because a replacement framing invites unsafe deployment. If AI is marketed as a low-cost substitute for scarce human care, older adults may be exposed to unmonitored emotional dependence, hallucinated advice, privacy violations, and inadequate crisis escalation. A relational augmentation framing instead treats GenAI as infrastructure: useful because it can make certain interactions more available and better organized, but legitimate only when embedded within human networks of responsibility.

This framing also protects the meaning-centered tradition from being flattened into generic positivity. Meaning-centered care does not require the older adult to reinterpret every loss as growth, nor does it treat suffering as a problem to be solved through optimistic language. It allows grief, ambivalence, regret, and finitude to be spoken. A GenAI system designed for this domain must therefore avoid premature reassurance. Its task is not to produce comforting answers, but to support careful inquiry: what the loss means, which relationships remain salient, what values continue to guide the person, and when human presence is necessary.

## 4 A sensing-narrating-connecting-governing framework

**Figure 1:**
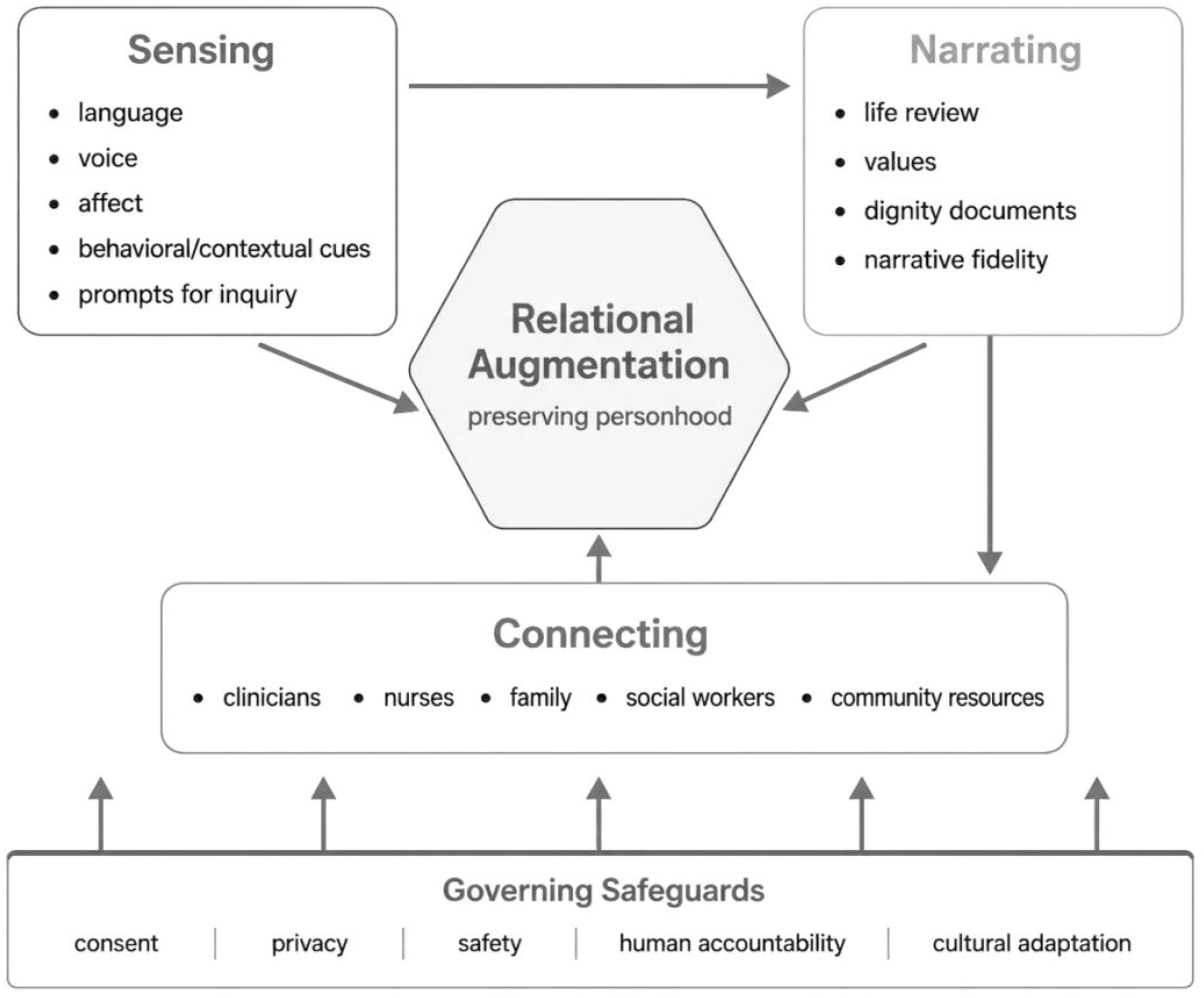
Conceptual model of GenAI as interactional infrastructure for meaning-centered care in later life. The model depicts four connected layers: Sensing, Narrating, Connecting, and Governing. Multimodal sensing identifies linguistic, vocal, affective, behavioral, and contextual cues relevant to existential distress and meaning-related needs. The narrating layer supports life review, dignity-preserving documentation, and values clarification. The connecting layer links AI-supported narratives to clinicians, nurses, social workers, family caregivers, and community resources. The governing layer surrounds all functions with safeguards for consent, privacy, hallucination control, crisis escalation, cultural adaptation, and human accountability. The central outcome is relational augmentation: preserving personhood by helping human systems listen, respond, and remain accountable.

We propose a four-layer framework for GenAI-supported meaning-centered care in later life: Sensing, Narrating, Connecting, and Governing (**Tables 1**, **2**; **Figure 1**). The framework is intended as a conceptual model for design, evaluation, and implementation rather than as a claim that any current system already meets these requirements.

**Table 1:** A Sensing-Narrating-Connecting-Governing framework for GenAI-supported meaning-centered care in later life.

Layer	Core function	Meaning-centered task	Key safeguards
Sensing	Detect language, voice, affective, behavioral, and contextual cues	Identify existential distress, loneliness, disrupted self-continuity, or meaning-related themes	Consent, transparency, data minimization, bias assessment, clinical validation
Narrating	Support life review and narrative organization	Elicit memories, values, roles, regrets, achievements, relationships, and legacy messages	Avoid false memories, distinguish user words from AI-generated text, preserve agency
Connecting	Link AI-supported narrative work to human care	Share summaries with clinicians, nurses, social workers, family caregivers, or community workers when appropriate	Human-in-the-loop review, escalation pathways, user control over sharing
Governing	Maintain safety, accountability, and equity	Define boundaries for use, referral, evaluation, and oversight	Hallucination control, crisis protocols, privacy protection, cultural adaptation, auditability

**Table 2:** Developmental horizon of the sensing-narrating-connecting-governing framework.

Layer	Plausible current support	Requires near-term development	Aspirational/not ready for autonomous use
Sensing	User-consented identification of explicit linguistic cues such as burden, loneliness, unfinished responsibilities, loss of role, or repeated requests for help; generation of review prompts for human staff.	Validation of multimodal cue models against clinician-rated or user-reported existential distress; calibration across culture, language, sensory impairment, and cognitive status.	Autonomous inference of meaning, spiritual distress, suicidality, neglect, or family conflict from passive signals without human interpretation.
Narrating	Prompt generation, timeline organization, draft summaries, legacy letters, family memory artifacts, and preservation of user-approved wording.	Interfaces that preserve provenance, dialect, emotional tone, and user authorship while supporting fatigue, low literacy, visual impairment, or mild cognitive vulnerability.	Automated delivery of dignity therapy, meaning-centered psychotherapy, or emotionally intensive life review without a trained human listener and clinical accountability.
Connecting	User-approved sharing of concise summaries with clinicians, nurses, social workers, family caregivers, or community workers.	Local workflows defining thresholds, reviewer roles, response time, documentation, and feedback loops after a flag is generated.	Fully automated routing, triage, or service allocation for emotionally or clinically complex concerns.
Governing	Consent, privacy protection, human review, hallucination checks, crisis protocols, data minimization, and refusal options.	Audit systems, adverse-event taxonomies, model monitoring, equity assessment, and alignment with applicable AI and medical-device or clinical decision-support rules.	Self-governing systems that determine clinical risk, care priorities, or family disclosure without accountable institutional oversight.

To avoid conflating present capability with future possibility, the framework should be read developmentally. Some functions, such as drafting life-review prompts or summarizing user-approved narratives, are already technically plausible when used in low-risk, human-supervised settings. Other functions, such as reliable detection of existential distress from multimodal signals, require further validation because current affective-computing and speech-language systems are better supported for domains such as depression, cognitive impairment, or general affect than for meaning-related inference. A third group of functions, including autonomous interpretation of spiritual distress or independent clinical escalation, should remain outside the scope of present deployment. The framework is therefore not a readiness claim; it is a map for bounded design and staged evaluation.

The four layers should be understood as mutually dependent. Sensing without narrating risks reducing the older adult to a stream of risk indicators. Narrating without connecting may create an emotionally intense but clinically isolated interaction. Connecting without governance may expose intimate life material to inappropriate sharing. Governance without attention to narrative may become a compliance exercise detached from the purpose of care. A useful system must therefore coordinate all four layers in a way that preserves agency and accountability.

### 4.1 Sensing: from symptom signals to meaning-related cues

The revised sensing layer is intentionally modest. Existing work in speech and language analysis provides useful precedents for detecting depression, cognitive impairment, or crisis-related signals, but these precedents do not establish that existential distress can be inferred reliably from multimodal data. In meaning-centered care, sensing should therefore begin with explicit, user-visible cues in conversation, such as repeated references to being a burden, social disconnection, unfinished responsibilities, loss of role, or fear that one’s life story will disappear. These cues should generate questions for further exploration or a recommendation for human review, not diagnostic labels. Meaning is context-dependent; a phrase that appears hopeless in one context may reflect spiritual acceptance or culturally specific humility in another.

For product design, this means that sensing outputs should be probabilistic, explainable, and modest. Instead of labeling a user as existentially distressed, a system might flag that the conversation contains repeated references to burden, social disconnection, or unfinished responsibilities, and then recommend human review or further exploration. Such a design keeps AI in an assistive role and reduces the risk that algorithmic inference becomes a substitute for clinical understanding.

### 4.2 Narrating: AI-assisted life review without narrative appropriation

The narrating layer is where GenAI has distinctive potential. LLMs can sustain open-ended conversation, ask follow-up questions, organize timelines, identify recurrent values, and transform fragmented recollections into drafts of letters, audio scripts, family memory books, or dignity documents. For older adults with limited literacy, visual impairment, or fatigue, voice-based and multimodal interfaces may lower the burden of participation. For those separated from family members, AI-assisted narrative artifacts may support intergenerational communication.

The digital translation of meaning-centered work is therefore best understood as partial and component-based. Digitally mediated tools may help with access, scheduling, prompt delivery, recording, transcription, summarization, and production of legacy documents. Marziliano and colleagues’ web-based adaptation of meaning-centered psychotherapy for caregivers illustrates that core therapeutic ideas can be translated into a digital format, but the adaptation still depends on careful intervention design, user support, and attention to loneliness and relational context (18). The AI contribution proposed here is narrower: it may support elicitation and organization of narrative material, while the tasks of witnessing, emotional pacing, interpretation of ambivalence, and response to risk remain human responsibilities.

At the same time, narrative co-construction creates ethical risks. AI-generated text may overwrite the person’s voice, embellish memories, introduce inaccuracies, or impose culturally generic meanings. The system must therefore preserve provenance: which words came directly from the older adult, which were summarized, and which were generated as suggested wording. Older adults should remain authors of their narratives, not raw material for algorithmic storytelling.

Practical design features can support this principle. Systems could display user-authored text and AI-suggested edits in separate layers, require explicit confirmation before any generated wording is saved, and offer options to preserve the original voice, dialect, or emotional tone. In family-facing outputs, the system should avoid smoothing away contradiction or sadness. A life story that is too polished may be less truthful and less meaningful than one that preserves the person’s own phrasing, hesitations, and priorities.

### 4.3 Connecting: preventing the AI island

A meaning-centered AI system should not become an isolated companion that absorbs distress without mobilizing care. The connecting layer links AI-supported conversations to human relationships and service pathways. With the older adult’s consent, summaries of meaning-related concerns could inform nurses, psychiatrists, general practitioners, social workers, spiritual care providers, or family caregivers. For example, repeated concerns about being a burden could trigger a human check-in; a completed dignity document could be discussed with family; unresolved grief could lead to referral for bereavement support.

For future implementation research, the connecting layer would need locally defined handoff rules rather than universal thresholds proposed by this Perspective. A low-acuity concern might be a user-approved summary for discussion at the next routine nursing or community-care contact. Repeated burden statements, unresolved grief, or persistent social withdrawal could prompt review by a named nurse, social worker, or primary-care clinician within a locally specified period. Statements suggesting self-harm, abuse, neglect, severe confusion, or acute deterioration would require immediate escalation according to existing clinical or safeguarding protocols. These examples are illustrative design considerations, not validated thresholds; they clarify where empirical workflow studies are needed.

This layer is central to the distinction between relational augmentation and replacement. The goal is not to keep the older person talking to the machine, but to use the machine to improve the timing, continuity, and personalization of human care.

The connecting layer is also where public mental health value emerges. Many older adults do not need specialist psychotherapy, but they do need someone to notice when loneliness becomes despair, when caregiving dependence becomes shame, or when an unfinished family conversation becomes a persistent source of distress. GenAI-supported summaries may help primary care and community teams identify these needs earlier, provided that summaries are short, clinically interpretable, and controlled by the older adult wherever possible.

### 4.4 Governing: care ethics as implementation infrastructure

The governing layer translates care ethics into operational requirements. Care ethics emphasizes attentiveness, responsibility, competence, responsiveness, and relational context (19). Applied to GenAI, this means that systems should be judged not only by accuracy or user satisfaction but by whether they sustain responsible relations of care. Governance should address at least five issues: hallucination risk, emotional dependency, privacy and secondary use of intimate narratives, unequal access among older adults with low digital literacy, and crisis escalation when suicidal ideation, abuse, neglect, or severe mental deterioration emerges.

Governance should be linked to the risk profile of the intended use. A tool that helps an older adult draft a family memory letter is closer to a low-risk supportive application, whereas a tool that flags possible self-harm, neglect, or clinical deterioration enters a higher-risk decision-support context. Emerging regulatory approaches, including the EU AI Act’s risk-based classification and guidance on AI-enabled clinical decision support, are useful because they ask whether the system informs, influences, or substitutes for professional judgment. In China, governance should also attend to the developing rules on algorithmic recommendation, generative AI services, personal information protection, and medical AI oversight. The practical implication is that meaning-centered GenAI should be treated as assistive infrastructure with documented intended use, human accountability, data minimization, audit trails, adverse-event review, and a clear right to refuse automation.

An assistive-not-autonomous principle is therefore essential (20). GenAI may provide prompts, summaries, and conversational scaffolding, but final responsibility for clinical decisions, risk management, and relational care must remain with accountable humans and institutions.

Governance should also include a right not to be automated. Some older adults may find AI-mediated conversations alienating, intrusive, or inconsistent with their understanding of care. Others may prefer family, spiritual care, peer support, or face-to-face counseling. Respecting refusal is not a barrier to innovation; it is a condition for ethically credible implementation. A meaning-centered system must be optional, transparent, and reversible.

## 5 Evaluation beyond model performance

The proposed framework requires a broader evaluation logic. Standard AI metrics such as accuracy, sensitivity, specificity, response relevance, user engagement, and completion rate remain useful, but they are insufficient for meaning-centered care. A system could score well on engagement while increasing emotional dependence, or produce fluent summaries while distorting the person’s voice. Conversely, a system that shortens a conversation because it appropriately triggers human follow-up may appear less engaging but clinically safer.

Evaluation should therefore include at least four domains. Existential outcomes may include the Meaning in Life Questionnaire, Schedule for Meaning in Life Evaluation, Patient Dignity Inventory, purpose-in-life measures, perceived burden scales, and qualitative assessments of continuity of self. Relational outcomes may include therapeutic alliance scales when a clinician is involved, perceived empathy and recognition, family communication quality, caregiver understanding of the older adult’s values, and network-of-care indicators such as whether a concern reached the appropriate staff member. Safety outcomes should include not only crisis events but also hallucinated or fabricated advice, inappropriate reassurance, distress after use, privacy breach, unwanted sharing, dependency, missed escalation, and inequitable access. Implementation outcomes should include review time, staff workload, completion and attrition, accessibility failures, cultural acceptability, proportion of AI-flagged concerns reviewed by humans, and proportion leading to a documented care action.

These domains also imply a staged research agenda, but the present article does not define a trial protocol. Early studies should examine usability, narrative fidelity, and acceptability among diverse older adults. Feasibility studies can then test whether AI-assisted life review can be integrated into routine nursing, primary care, palliative care, or community mental health workflows. Before controlled trials, future empirical work should establish more than user satisfaction: acceptable usability for older adults with sensory, literacy, and cognitive variation; evidence that AI-generated summaries preserve the user’s meaning and do not introduce false memories; low and monitored rates of adverse responses; workable human-review and escalation procedures; manageable workflow burden; and participant understanding of consent, data use, and refusal options. Harm monitoring could include session-level adverse-event logs, user distress checks before and after use, staff review of flagged conversations, rapid reporting of crisis or privacy events, and independent oversight when emotionally intensive or clinically risky use cases are tested.

## 6 Implementation priorities for clinical and public mental health settings

For GenAI-supported meaning-centered care to become clinically useful, implementation must move beyond prototype enthusiasm. First, systems should be designed for specific settings: community mental health, primary care, long-term care facilities, palliative care, dementia care, and family caregiver support each require different boundaries. Early work adapting meaning-centered psychotherapy into web-based support and broader reviews of conversational agents in health care suggest that digital translation is feasible, but also that implementation quality and emotional depth vary substantially (18, 21). Second, workflows should specify who reviews AI-generated summaries, how often, and what actions follow. Third, evaluation should include outcomes that reflect meaning-centered goals: meaning in life, dignity, existential well-being, loneliness, perceived burden, continuity of self, therapeutic alliance, caregiver communication, and service linkage.

Implementation should draw on existing digital health and assistive-technology frameworks rather than treat GenAI as an exception. Participatory design, iterative feasibility testing, accessibility evaluation, engagement analysis, workflow integration, and post-deployment monitoring are all relevant. What needs additional attention in meaning-centered GenAI is the handling of biographical material: provenance of personal words, preservation of voice, consent for family or clinical sharing, escalation after existential or relational risk cues, and protection against turning intimate narrative into unreviewed data exhaust.

Conversational-agent research with older adults also cautions against assuming that availability equals acceptability. Older users may value companionship and convenience, but engagement is shaped by trust, perceived intrusiveness, sensory and motor accessibility, conversational breakdowns, digital literacy, family attitudes, and the burden of repeated use. Attrition and non-use should be treated as clinically meaningful implementation outcomes rather than as mere technical failures. A meaning-centered system that is difficult to hear, read, control, or exit may undermine dignity even if its language model performs well.

Fourth, cultural adaptation should be treated as a core design requirement rather than a *post-hoc* translation task. Studies of smart health care and electronic personal health records among older adults in China indicate that digital health benefits depend on trust, interface design, access, and social support rather than availability alone (22, 23). In many Asian and collectivist contexts, meaning may be organized through family continuity, filial relationships, contribution to descendants, moral duty, and collective memory. AI systems that rely on individualistic assumptions about self-expression may fail to elicit these sources of meaning or may misinterpret indirect communication. Fifth, older adults should be involved in co-design, especially those with sensory impairment, low digital literacy, cognitive vulnerability, rural residence, or limited social support. Without this, GenAI may widen rather than narrow the digital meaning divide.

Finally, clinical product implementation should use staged validation. Early studies can assess usability, acceptability, and narrative fidelity. Subsequent trials can examine whether AI-assisted life review improves meaning-related outcomes compared with usual digital support or human-only low-intensity interventions. Real-world studies should evaluate safety events, escalation accuracy, workflow burden, inequity, and long-term dependency. Existing work on AI-assisted crisis detection and conversational agents for older adults illustrates two complementary lessons: risk signals can be operationalized in service workflows, and relationship-sensitive interface design matters for sustained engagement (24, 25).

## 7 Discussion

GenAI creates a genuine opportunity for late-life mental health, but only if the field resists the wrong metaphor. The metaphor of the “AI therapist” focuses attention on replacement: can the machine imitate empathy, deliver therapy, and reduce workforce costs? A better metaphor is interactional infrastructure. Infrastructure does not replace care; it shapes the conditions under which care becomes timely, continuous, personalized, and accountable.

For older adults, this infrastructure could make meaning-centered conversations more accessible. It could help clinicians notice existential distress earlier, help families receive preserved stories, help care teams understand what matters to the person, and help public mental health systems move beyond a deficit model of aging. At its best, GenAI could support the preservation of personhood in systems that are often too hurried to listen.

The same technology can also fail in predictable ways. It can simulate intimacy without responsibility, produce fluent but inaccurate narratives, collect deeply personal data without adequate protection, or shift the burden of care from institutions to vulnerable individuals. These risks are not peripheral; they define the ethical boundary of the field.

These risks are visible in digital mental health more broadly. Inappropriate responses can intensify distress; fluent but generic reassurance can close down disclosure; unmonitored companionship can foster dependency; and systems designed for digitally confident users can widen access inequities. In older adults, these risks may be amplified by bereavement, cognitive impairment, sensory limitations, social isolation, and deference to perceived authority. These harms do not preclude GenAI use, but they make human supervision, refusal options, adverse-event monitoring, and equity assessment central rather than optional.

We therefore propose that future work on GenAI and older adults’ mental health adopt three commitments. First, design should begin from meaning, dignity, and relational continuity, not only from symptoms and service efficiency. Second, GenAI should be implemented as assistive and human-supervised, with clear escalation and accountability pathways. Third, evaluation should include existential, relational, equity, and workflow outcomes alongside technical performance.

There are several limitations to this perspective. The framework is not based on a systematic review, and the evidence base for GenAI-supported meaning-centered care remains early. Many claims are therefore hypothesis-generating rather than practice-changing. In addition, the feasibility of the model will vary across settings with different workforce capacity, digital infrastructure, privacy regulation, and cultural expectations about aging, family, and disclosure. These limitations reinforce rather than weaken the need for careful implementation research. The field should not move directly from technical possibility to unsupervised deployment.

Two rapidly developing sources on LLMs and speech-language AI in mental health were consulted during manuscript preparation as early online or preprint/online-first literature; the reference list has been updated to reflect their current publication status where available. This timing is relevant because the empirical base for GenAI in mental health is changing quickly and should be interpreted as emerging rather than settled.

Meaning-centered care reminds us that late-life mental health is not only about reducing distress; it is also about preserving the possibility that a person can still narrate a coherent, valued, and connected life. GenAI will contribute to that aim only when it helps human systems listen better.

## Data Availability

The original contributions presented in the study are included in the article/supplementary material. Further inquiries can be directed to the corresponding author.
